# An Inter-Networking Mechanism with Stepwise Synchronization for Wireless Sensor Networks

**DOI:** 10.3390/s110908241

**Published:** 2011-08-25

**Authors:** Hiroshi Yamamoto, Naoki Wakamiya, Masayuki Murata

**Affiliations:** Graduate School of Information Science and Technology, Osaka University, 1-5 Yamadaoka, Suita, Osaka 565-0871, Japan; E-Mails: wakamiya@ist.osaka-u.ac.jp (N.W.); murata@ist.osaka-u.ac.jp (M.M.)

**Keywords:** wireless sensor network, synchronization, pulse-coupled oscillator model

## Abstract

To realize the ambient information society, multiple wireless networks deployed in the region and devices carried by users are required to cooperate with each other. Since duty cycles and operational frequencies are different among networks, we need a mechanism to allow networks to efficiently exchange messages. For this purpose, we propose a novel inter-networking mechanism where two networks are synchronized with each other in a moderate manner, which we call stepwise synchronization. With our proposal, to bridge the gap between intrinsic operational frequencies, nodes near the border of networks adjust their operational frequencies in a stepwise fashion based on the pulse-coupled oscillator model as a fundamental theory of synchronization. Through simulation experiments, we show that the communication delay and the energy consumption of border nodes are reduced, which enables wireless sensor networks to communicate longer with each other.

## 1. Introduction

The ambient information society is the concept and framework where intelligent environment detects, reasons, and satisfies overt and potential demands of people without their interaction [1–3]. In the ambient information society, people do not need to be aware of existence of networked information devices embedded in the environment. They do not need to intentionally access a network to control the environment to make it comfortable and satisfy their demands. Instead, the embedded network controls the environment and provides personalized information services to a user taking into account time, place, occasion, and person.

To realize the ambient information society, networks deployed and operating in the same environment must cooperate with each other in exchanging information, sharing information, and controlling each other. For example, a person has a wireless body area network which consists of vital sensors, accelerometers, PDA, and other devices. On the other hand, a room has embedded wired and wireless networks which consist of sensors and actuators for environmental control. Intelligent home appliances also constitute embedded networks. When the person enters the room, those networks should cooperate with each other for smart environmental control. However, in general, those devices organize different and independent networks operating on different control policies. Therefore, to let the room provide the person with a comfortable environment, we need a mechanism for different networks to smoothly and dynamically connect and share their information. However, it is not a trivial task.

In general, wireless sensor networks adopt a sleep scheduling or duty cycling mechanism to save energy. Operational frequencies, that is, frequencies that they wake up and resume operation, are different among networks depending on application’s requirement and characteristics of devices. For example, an air conditioner would obtain and use the temperature information every minute to adjust its thermostat. On the other hand, devices to detect locations of people have to report their detection result very frequently at an order of seconds. When they want to exchange information among them for intelligent control of room temperature to intensively regulate the temperature around a person in the room, a node belonging to the location detection system has to stay active in order to wait for a node belonging to the thermal management system to wake up in transmitting a message. Even when an energy-efficient MAC protocol such as S-MAC [4] and X-MAC [5] is used, such communication consumes the substantial energy at the former node and thus risks energy depletion.

There are several proposals on dynamic composition of multiple networks [6,7]. In [6], they consider a mechanism for overlay networks to dynamically compose a hierarchical structure by two types of composition schemes, *i.e.*, absorption and gatewaying. In [7], cooperation between wireless networks is accomplished by organizing an overlay network by connecting gateway nodes belonging to different wireless networks. Although they can be applied to ambient information networking to some extent, they have a major problem that they do not take into account the difference in operational policies, more specifically, operational frequencies of different wireless sensor networks.

ZigBee [8], a standard protocol for wireless sensor networks, also provides interconnecting schemes such as PAN bridge, PAN marge and Peer-to-Peer, which enable a ZigBee Personal Area Network (PAN) to communicate with other PANs [9]. However, they also do not take into account the difference of the operational frequency. Although a bridge node can mediate communication among PANs with different operational frequencies, the bridge node consumes much energy and it would shorten the lifetime of PANs.

To address the problem, we propose stepwise synchronization between wireless sensor networks for smooth and moderate inter-networking, where sensor nodes located near the border of two networks adjust their operational frequencies to bridge the gap in their intrinsic operational frequencies [10]. Since only nodes near the border change their operational frequency, the remaining nodes can keep their frequency and thus energy consumption in inter-networking can be reduced. The stepwise synchronization is self-organized based on a nonlinear mathematical model of synchronization of oscillators, called the pulse-coupled oscillator (PCO) model [11]. The PCO model describes emergence of synchronization in a group oscillators with different frequencies by mutual interactions through stimuli. By adopting the PCO model to scheduling, operational frequencies of nodes can be appropriately adjusted without any centralized control in wireless sensor networks [12–15]. In our mechanism, we strengthen the degree of entrainment at border nodes to intensively shift the operational frequency toward that of the other network while the degree of entrainment is weakened as the distance to the border increase. As a result, the operational frequencies of nodes near the border are adjusted to somewhere between the original operational frequencies of wireless sensor networks. In our preliminary work [10], we propose the concept of our stepwise synchronization and show results of an early proof-of-concept mechanism in comparison with the case where each of networks keeps its intrinsic operational frequency. In this paper, we design details of our proposal such as a mechanism for nodes to autonomously adjust the degree of entrainment in accordance with the distance to the border. Furthermore, we evaluate the robustness and adaptivity of our proposal and the influence of parameter setting.

The rest of this paper is organized as follow. First in Section 2, we explain the pulse-coupled oscillator model. Next in Section 3, we describe the details of our proposal. In Section 4, we show and discuss results of our simulation experiments. Finally, we conclude the paper in Section 5.

## 2. Pulse-Coupled Oscillator Model and Synchronization

A pulse-coupled oscillator model is a mathematical model which explains synchronized flashing of a group of fireflies [11]. It is considered that a firefly maintains a biological timer, based on which it intermittently flashes. The flashing frequency depends on its intrinsic timer frequency, which could be different among individuals. However, when fireflies form a group, they begin to flash in synchrony. A mechanism of biological synchronization is explained as follow. When a firefly observes a flash of another firefly, it is stimulated and its timer advances by a small amount. Because of nonlinearity in timer or stimulus, by repeatedly stimulating each other, their timers begin to expire synchronously, then flash at the same time. Among PCO models [11,16,17], in this paper we use the model proposed in [11].

In the PCO model [11], oscillator *i* maintains phase *ϕ_i_* (0 ≤ *ϕ_i_* ≤ 1) of a timer and state *x_i_* (0 ≤ *x_i_* ≤ 1) given by a function of phase. The dynamics of phase *ϕ_i_* is determined by the following differential equation:

(1) $$\frac{d\phi_{i}}{dt}=F_{i}$$

where *F_i_* (*F_i_* *>* 0) stands for the intrinsic timer frequency of oscillator *i*. State *x_i_* is determined from phase *ϕ_i_* by the following monotonically increasing nonlinear function,

(2) $$x_{i}=\frac{1}{b}\text{ln}\left[1+\left(e^{b}-1\right)\phi_{i}\right]$$

where *b* (*b >* 0) is a dissipation parameter that dominates the rate of synchronization.

When phase *ϕ_i_* and state *x_i_* reach 1, oscillator *i* fires and both phase *ϕ_i_* and state *x_i_* go back to 0. When an oscillator fires, the oscillator stimulates oscillators that are coupled with the firing oscillator. If oscillator *j* is stimulated by oscillator *i* at time *t*, oscillator *j* increases its state *x_j_* by a small amount *ε* and phase *ϕ_j_* changes accordingly as

(3) $$x_{j}(t^{+})=B(x_{j}(t)+\varepsilon )$$

where

$$B(x)=\{\begin{array}{l} x\;(0\leq x\leq 1)\\ 0\;(x<0)\\ 1\;(x>0) \end{array}$$

and

(4) $$\phi_{j}(t^{+})=\frac{e^{{\mathrm{bx}}_{j}(t^{+})}-1}{e^{b}-1}$$

When state *x_j_*(*t*^+^) and phase *ϕ_j_*(*t*^+^) reach 1 by being stimulated, oscillator *j* also fires. Once oscillator *j* fires by being stimulated by oscillator *i*, oscillator *j* continually fires by being stimulated by oscillator *i*, if *F_i_* is greater than or equal to *F_j_*. If *F_i_* is less than *F_j_*, oscillator *i* continually fires by being stimulated by oscillator *j*. At this time, oscillators *i* and *j* are considered synchronized. To avoid overshoot and instability, an oscillator is not stimulated by two or more oscillators at the same time, and an oscillator is not stimulated at the time when it fires.

**Figure 1.**
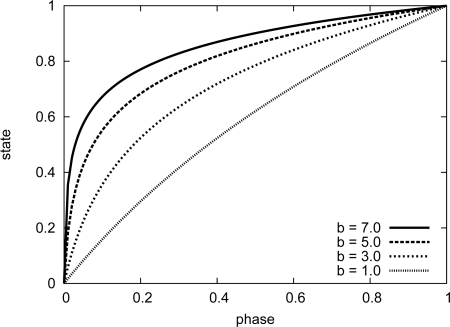
Effect of changing *b* on relationship between state and phase.

Figure 1 shows how dissipation parameter *b* affects the relationship between state *x* and phase *ϕ*, where *b* is changed from 1.0 to 7.0. As can be seen in Figure 1, when *b* is set at a small value, e.g., 1.0, the amount of phase shift on receiving a stimulus is almost the same despite the timing that the stimulus is received. On the other hand, in the case that *b* is large, e.g., 7.0, a stimulated oscillator changes its phase by a large amount when its state value is large.

**Figure 2.**
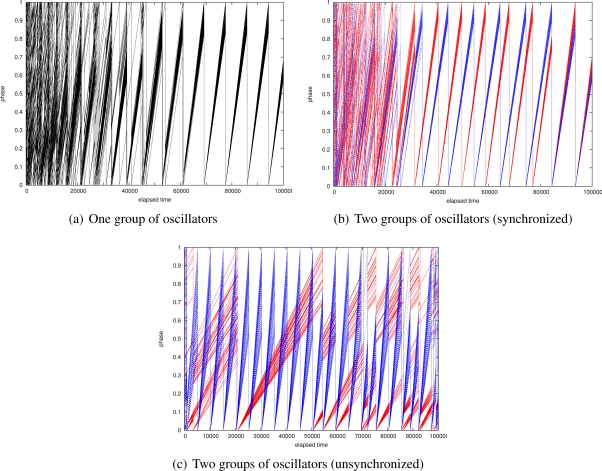
Phase transition in numerical analysis.

In Figure 2, we show results of numerical analysis for three different scenarios. First, Figure 2(a) shows phase transition of 100 oscillators arranged in a 10 × 10 grid. Intrinsic frequency of each oscillator is randomly chosen within the range of [0.9, 1.1]. Initial phase *ϕ* is also chosen at random. An oscillator is coupled with four neighbors located in up, right, down and left of the oscillator and stimuli are never lost and always received by the four neighbors. There is no delay in stimuli propagation and phase and state change. *b* and *ε* are set at 3.0 and 0.1, respectively. At first, phases are different among oscillators. The increasing rates of phase are also different among oscillators due to the difference of intrinsic frequency. As time passes, timings of firing gradually get closer by stimulating each other. Finally, all oscillators become synchronized and fire at the same time.

Then, we show how two groups of oscillators whose intrinsic frequencies are different get synchronized to the same operational frequency. As in the above example, 100 oscillator are arranged in a 10 × 10 grid. The initial phase is chosen at random, and *b* and *ε* are set at 3.0 and 0.1, respectively. Half of oscillators in the left forming a 5 × 10 grid set their intrinsic frequencies within the range of [0.9, 1.0] at random, and they belong to group 1. The other oscillators set their intrinsic frequency within the range of [1.0, 1.1] at random, and they belong to group 2. During the first four-fifths of simulation time, oscillators are not stimulated by oscillators belonging to the other group. That is, there is no coupling among oscillators of different groups. During the last one fifth of simulation time, oscillators at the border are coupled with each other so that they can stimulate each other. As can be seen in Figure 2(b), during the first four-fifths, oscillators belonging to the same group identified by the same color get synchronized and oscillators belonging to different groups fire at different timings. As inter-group communication is allowed at time 80,000, two groups become integrated and all oscillators fire at the same time and same frequency. The operational frequency of the unified group is dominated by the highest frequency of intrinsic frequencies of all oscillators, *i.e.*, about 1.1.

Finally, we show a case that two groups of oscillators cannot accomplish synchronization for a big difference in operational frequencies. From the above setting, we change the ranges of operational frequency to [0.2, 0.3] for group 1 and [1.0, 1.2] for group 2. An inter-group communication is allowed at time 50,000. An obtained result is shown in Figure 2(c). As can be seen, the global synchronization cannot be accomplished and synchronization in each group is even lost by being disturbed by stimuli from the other group.

**Figure 3.**
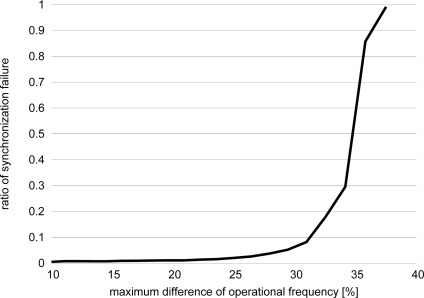
Ratio of synchronization failure against maximum difference in operational frequency.

To see how difference in operational frequency affects synchronization, Figure 3 illustrates the relationship between the difference and the ratio of synchronization failure. We distribute 100 oscillators randomly in a 100 × 100 region. Each oscillator is coupled with oscillators within a 25 radius. Stimuli are never lost and received by coupled oscillators with no delay. The difference in operational frequency on the X-axis is changed by adjusting the range of operational frequency, between which an intrinsic operational frequency of each oscillator is randomly chosen. More specifically, the upper limit is set at 1.1 and the lower limit is changed from 1.0 to 0.8. When the range is [1.0, 1.1], the difference is given as (1.1−1.0)/1.0 = 0.1. Initial phase is set at random, and *b* and *ε* are set at 3.0 and 0.1, respectively. We conducted 10,000 simulation runs for each of operational frequency settings. The Y-axis shows the ratio that the global synchronization cannot be accomplished until the end of simulation run, *i.e.*, 100,000 simulation time units, among 10,000 simulation runs. In Figure 3, when the maximum difference in operational frequency is small, the global synchronization can be accomplished with high probability. However, when the maximum difference is greater than 30%, the ratio of the synchronization failure suddenly increases. The result supports a need for a stepwise synchronization mechanism for wireless sensor networks adopting PCO-based synchronization mechanism [12] to be interconnected without too much burden on border nodes.

**Figure 4.**
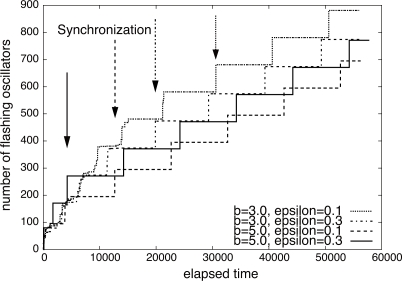
Cumulative number of flashing oscillators.

Not only operational frequencies but parameters of PCO also influence synchronization. In Figure 4, we show how the cumulative number of flashing oscillators changes with different parameters *b* and *ε* against time. 100 oscillators are arranged in a 10 × 10 grid. Each oscillator is coupled with four neighbors. The operational frequency is identical among oscillators and their initial phase is set at random. The height of stepwise increase in the number corresponds to the number of oscillators simultaneously flashing. As indicated by arrows, the time when a group of oscillators reach synchronization and begins to flash in synchrony is about 30,000 with “*b* = 3.0, *ε* = 0.1”, about 20,000 with “*b* = 3.0, *ε* = 0.3”, about 13,000 with “*b* = 5.0, *ε* = 0.1”, and about 4,000 with “*b* = 5.0, *ε* = 0.3”, respectively. When we adopt larger *b* and *ε*, the speed of synchronization apparently increases. This fact is a main source of our idea to achieve the stepwise synchronization. Much larger parameters further accelerate synchronization, but the stability deteriorates [12]. Although delay is not taken into account in these figures, it is known that the synchronization is accomplished in the environment with loss and delay of stimuli [18,19].

## 3. Inter-Networking Mechanism with Stepwise Synchronization

In this section, we propose an inter-networking mechanism with stepwise synchronization based on pulse-coupled oscillator model. First, we describe a targeted scenario and how we apply the PCO model to synchronization in wireless sensor networks, and then, we explain our stepwise synchronization-based inter-networking mechanism.

### 3.1. Target Scenario

In this paper, we assume that two or more wireless sensor networks co-exist in the region, *i.e.*, field or room. Each wireless sensor network is composed of nodes designated for a specific application, such as temperature control, health monitoring, home security, *etc.* There can exist a server or central control unit, e.g., home server, which gather sensor information and control nodes. Each node is capable of wireless communication with other nodes within the radio range independently of wireless sensor networks to which they belong. Each node adopts duty cycling with which it switches between two states, *i.e.*, active and sleep. An operational interval of node is the sum of active and sleep periods in one duty cycle. Operational intervals are identical among nodes belonging to a wireless sensor network and they are synchronized. For example, in a wireless sensor network to monitor temperature of a room at intervals of one minute, all nodes in the network simultaneously wake up every minute, obtain and report temperature, and go back to sleep. An operational frequency of node is defined as a reciprocal of an operational interval. Synchronization of nodes is accomplished within a wireless sensor network by a PCO-based synchronization mechanism such as [12]. We consider a scenario where multiple wireless sensor networks with different operational frequencies exchange sensor information and control messages with each other for integrated and intelligent services as stated in Section 1.

### 3.2. PCO Based Synchronization in Wireless Sensor Networks

In applying the PCO model to synchronization, a wireless sensor node corresponds to an oscillator. It stimulates neighbor nodes in the range of radio signals by broadcasting a message. A message is used for both of synchronization and data communication with such a mechanism where control messages for synchronization are embedded in messages for sensor data [20].

Node *i* maintains state *x_i_* and phase *ϕ_i_* as variables of a timer of frequency *F_i_* and calculates its new state and phase at regular intervals, e.g., at the granularity of timer. When state *x_i_* and phase *ϕ_i_* reach 1, node *i* sets both state *x_i_* and phase *ϕ_i_* at 0 and tries to broadcast a message which informs neighbor nodes that the node fires. Since a wireless channel is the shared medium, there is possibility that broadcasting is delayed to wait for the channel to become available. However, from our previous experiments, the influence of delay on synchronization is negligible [18]. When a node receives a broadcast message, it is stimulated. The stimulated node, say node *j*, increments its state *x_j_* by a small amount *ε* by Equation (3) and calculates new phase $\phi_{j}^{+}$ based on the new state $x_{j}^{+}$ by using Equation (4). If the new state $x_{j}^{+}$ and new phase $\phi_{j}^{+}$ reach 1, node *j* also fires and broadcasts a message. Since duty cycling is adopted on a node, only neighboring nodes that are awake can receive stimuli. Details of integration of duty cycling and the PCO model will be given later.

### 3.3. Overview

**Figure 5.**
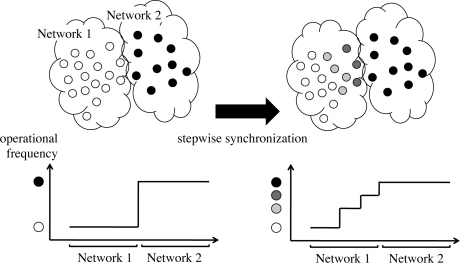
Stepwise synchronization.

Now we propose a stepwise synchronization-based inter-networking mechanism. As an example, in Figure 5, two wireless sensor networks with different operational frequencies are adjacent, and they attempt to cooperate. When we couple border nodes to let them stimulate each other, two wireless sensor networks will be unified to a single network with the operational frequency identical to the faster one. However, it sacrifices too much energy of nodes belonging to a slower network by forcing them to wake up more frequently. Even if such unification and synchronization are allowed, there is limitation on the difference in operational frequencies in achieving the global synchronization. As shown in Figures 2(c) and 3, it is hard for two networks whose operational frequencies are different by more than 35% to be fully synchronized. Therefore, we need a new mechanism to accomplish stepwise synchronization where only a part of network is involved in the synchronization and those networks with largely different operational frequencies can be synchronized. For this purpose, we adjust the degree of entrainment in accordance with the distance to the border. We focus on the fact that the dissipation *b* and the stimulus *ε* influence the degree of entrainment and the speed of synchronization (see Figure 4).

In our proposal, we set larger values of *b* and *ε* at nodes located at the border of wireless sensor networks to strengthen entrainment and shift the operational frequency much. By receiving stimuli from the other network, nodes located at the border of wireless sensor networks actively changes their operational frequencies for larger parameters. Then smaller values are applied to nodes as the distance to the border becomes larger. Nodes distant from the border of wireless sensor networks are also entrained by receiving stimuli from nodes located at the border, but the impact is smaller for smaller parameters and thus their operational frequencies stay rather closer to the original frequency. Consequently, we observe a stepwise change in operational frequencies around the border of two networks as illustrated in Figure 5. Such stepwise synchronization can bridge the large gap in operational frequencies which cannot be overcome by the PCO model alone.

**Figure 6.**
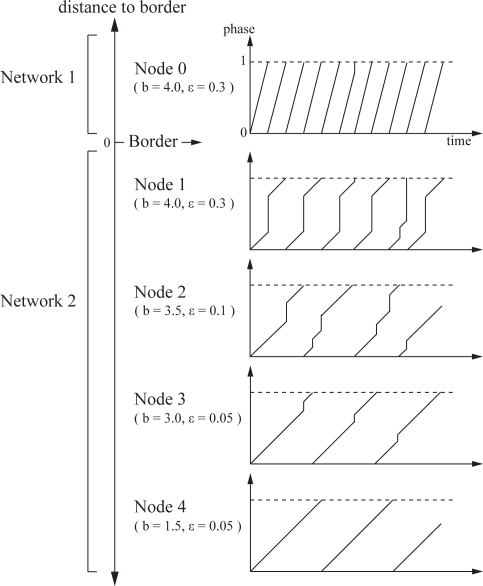
Phase transition in PCO based stepwise synchronization.

Figure 6 illustrates phase transition when two wireless sensor networks adopt our stepwise synchronization. Note that the intrinsic operational frequency of Network 1 is faster than that of Network 2. In Figure 6, nodes located at the border of networks apply larger values to parameters *b* and *ε*, e.g., “*b* = 4.0, *ε* = 0.3”. Due to the larger values of *b* and *ε*, Node 1 increases its state largely when it receives a stimulus and the interval between two firing timings, *i.e.*, the operational interval, become shorter. The operational interval of Node 2 also become shorter by receiving stimuli from Node 1 more frequently than the original operational frequency. However the degree of change in the operational interval is smaller than Node 1, because the entrainment is weaker for the smaller parameters *b* and *ε*. As the distance to the border increases, the strength of entrainment becomes smaller and thus the degree of change in the operational frequency becomes smaller.

### 3.4. Node’s Behavior

**Figure 7.**
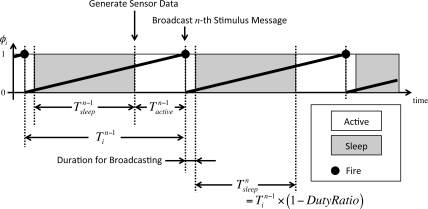
Duty cycling in proposal.

Figure 7 shows the duty cycling in our proposal. Node *i* maintains state *x_i_* and phase *ϕ_i_* of a timer of frequency *F_i_*. The phase automatically advances and the state accordingly changes independently of whether it is awake or sleeping. When state *x_i_* and phase *ϕ_i_* reach 1, node *i* sets both state *x_i_* and phase *ϕ_i_* at 0 and tries to broadcast a stimulus message. After the duration required to broadcast a stimulus message, node *i* goes to sleep for $T_{\mathrm{sleep}}^{n}=T_{i}^{n-1}\times \left(1-\mathrm{DutyRatio}\right)$ independently whether it could successfully broadcast a stimulus message or not. $T_{i}^{n-1}$, called operational interval, is defined as the duration from the *n −* 1-th firing timing to the *n*-th firing timing. *Duty Ratio* is the duty ratio parameter which is determined in advance. Although a sleeping node does not receive any stimulus message, there is a case that the state and phase occasionally reach 1. In such a case, a node wakes up to broadcast a stimulus message and after broadcasting it immediately goes to sleep. When $T_{\mathrm{sleep}}^{n}$ has passed, the node wakes up and becomes capable of sending and receiving messages for the duration of $T_{\mathrm{active}}^{n}$.

If a node has received messages from other networks, it, *i.e.*, a border node, adjusts *b* and *ε* at *b_max_* and *ε_max_*, where *b_max_* and *ε_max_* are maximum values of *b* and *ε*, respectively. A stimulus message emitted by a border node contains updated parameters *b* and *ε*, and attenuation coefficients *A_b_* (0 < *A_b_* < 1) and *A_ε_* (0 < *A_ε_* < 1) .

When node *i* receives a stimulus message in its active period, it calculates its new *b* and *ε* by following equations.

$$\begin{array}{l} b_{i}^{+}=\text{max}(A_{b}\times b_{\mathrm{stim},}\;\;b_{\min})\\ \varepsilon_{i}^{+}=\text{max}(A_{\varepsilon }\times \varepsilon_{\mathrm{stim}}\;\;\varepsilon_{\min}) \end{array}$$

where $b_{i}^{+}$ and $\varepsilon_{i}^{+}$ stand for new *b* and *ε. b_stim_* and *ε_stim_* stand for values of *b* and *ε* in the received stimulus message. *b_min_* and *ε_min_* are minimum values of *b* and *ε*. When a node receives two or more stimulus messages that have different values of *b* and *ε*, it uses the largest values among them. Then, node *i* calculates its new state and phase by Equations (3) and (4). Finally, the updated $b_{i}^{+}$ and $\varepsilon_{i}^{+}$, *A_b_* and *A_ε_* are embedded as *b_stim_*, *ε_stim_*, *A_b_*, and *A_ε_* in stimulus messages emitted by the node. If the new state and new phase reach 1, node *i* broadcasts a stimulus message and goes to sleep.

A border node stops embedding *b*, *ε*, *A_b_* and *A_ε_* in a stimulus message, if it has not received any messages from other networks for a certain period of time to notify other nodes of the end of cooperation. By receiving stimulus messages without *b*, *ε*, *A_b_* and *A_ε_*, other nodes also stop embedding these information in stimulus messages.

We should note here that our proposal requires simple floating point arithmetic and as such the computational complexity is not high. Furthermore, each node broadcasts a stimulus message, which can be embedded in a normal message for, e.g., data gathering, only once an operational interval. Thus, the communication overhead is low. Therefore, our proposal can be implemented on a low cost and low performance sensor node and easy to deploy.

## 4. Performance Evaluation

### 4.1. Simulation Settings

**Figure 8.**
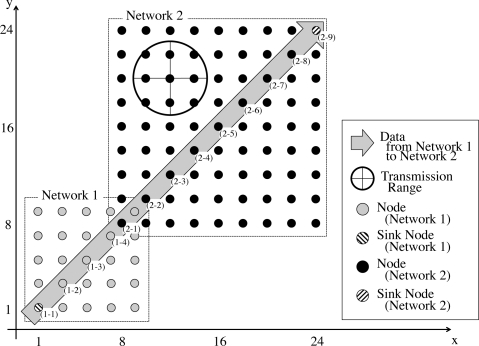
Node layout in simulation.

We arranged 106 nodes in a 24 × 24 area as shown in Figure 8. In Figure 8, nodes in the lower-left area belong to Network 1 and the others do Network 2. Therefore, each of networks has four border nodes located in the overlapping area. A circle shown in Figure 8 illustrates the communication range of the node centered at the circle. Parameters are set as summarized in Table 1. In addition to duty cycling based on the PCO-model, we further adopt duty cycling on the MAC layer, that is X-MAC [5]. In Table 1, *S_pre_*, *S_ack_*, *S_stim_* and *S_data_* stand for durations that a node sends a Short Preamble, an ACK, a stimulus message and a data packet, respectively. We assumed that 2 wireless sensor networks operating at intervals of 10 s and 60 s co-exist and share sensor data between them. Nodes belonging to Network 1 set their intrinsic operational frequencies within the range of [0.100, 0.101] at random, and nodes belonging to Network 2 set their intrinsic operational frequencies within the range of [0.01700, 0.01717] at random. The operational interval between successive broadcasting is about 10 s in Network 1 and about 60 s in Network 2. Initial phases are set at random. The duty ratio is set at 0.3 at all nodes. The simulation time is 50,000 s in each simulation run.

In our simulation, the sink node of Network 1 sends a data message to the sink node of Network 2 by using multihop unicast communication once per 10 operational cycles. Data messages take the shortest path to the receiver node following the diagonal of the networks as shown in the figure. When a node between the sink nodes is active and receives a data message, it immediately tries sending the message to a next-hop node. It transmits preambles until it receives an ACK from the next-hop node, even after the end of the PCO-based active period, *i.e.*, expiration of timer. When the transmission of the data message is completed after the expiration of timer, the node moves to the sleep state.

For the purpose of evaluation of energy consumption, we assume that each node is equipped with an Atmel ATmega 128L processor, a Texas Instruments CC2420 radio chip and two AA batteries. The details of energy consumption model is summarized in Table 2.

### 4.2. Operational Frequency

**Figure 9.**
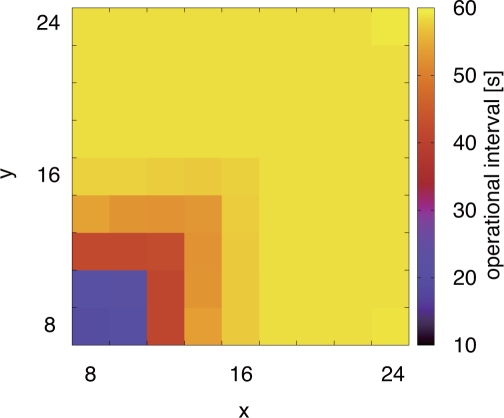
Operational interval in stepwise synchronization (Network 2).

We first confirm that the stepwise synchronization is accomplished. Figure 9 shows how nodes in Network 2 (slower network) adapt their operational frequencies. Each square corresponds to a node and the color shows the average operational interval adapted by a node during the last 10,000 s of simulation time. In this figure, we see that the operational interval of nodes at the border, *i.e.*, four nodes located lower-left of the network, becomes about 20 s, closer to the operational interval of Network 1. On the other hand, the operational interval of other nodes become longer than that of those border nodes as the distances to the border become larger. As a result, the stepwise change in operational frequency emerges in accordance with the distance to the border.

**Figure 10.**
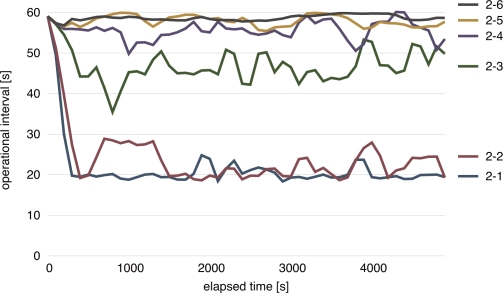
Temporal change of operational interval in stepwise synchronization.

Figure 10 shows the temporal change of operational intervals adapted by Nodes (2-1), (2-2), (2-3), (2-4), (2-5), and (2-6) during first 5,000 s. In this figure, the operational interval averaged in a 100 s time window is shown. As can be seen in Figure 10, operational intervals of Node (2-1) and (2-2), *i.e.*, border nodes, are shorten to about 20 s immediately after the beginning of the stepwise synchronization. By being stimulated by Node (2-2), the operational interval of Node (2-3) decreases to about 45 s on average and it contributes to filling the difference in operational intervals between border nodes and other nodes. The operational interval of other nodes slightly change but they are still closer to the original intervals as the distance to the border increases. A reason why operational intervals fluctuate is that stimulus messages are occasionally lost. A loss of a stimulus message prevents a node from being stimulated and adjusting an operational interval. Nevertheless, we can see the stepwise condition of operational intervals is successfully accomplished.

### 4.3. Communication Delay and Energy Consumption

We next compare two scenarios, where both networks keep their intrinsic frequencies denoted as “independent”, and our proposal is adopted denoted as “proposal”. As performance measures, we use communication delay which is defined as the duration between the time when a node begins to send preambles for transmission of a data message and the time when a node receives an ACK for message reception.

**Figure 11.**
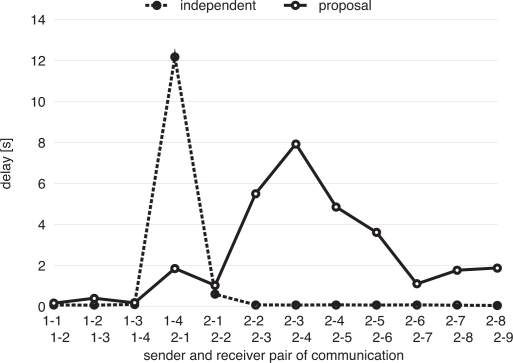
Communication delay of each hop.

Figure 11 shows the median of the communication delay of all data messages transmitted in simulation runs. In Figure 11, “1-1, 1-2” corresponds to the communication delay from Node (1-1) to Node (1-2), for example. Those nodes from Node (1-1) to Node (1-4) belong to Network 1 (faster network), and those from Node (2-1) to Node (2-9) belong to Network 2 (slower network). Node (1-4), Node (2-1) and Node (2-2) are nodes located at the border of networks. In Figure 11, in the case of “independent”, although all communication delays between nodes belonging to the same network are quite small, communication delay between nodes located at the border is large at about 12.2 s. It is because Node (1-4) has to wait for Node (2-1) located at the border of Network 2 to wake up in transmitting a data message. On the other hand, in the case that our proposal is adopted, communication delay at the border node becomes small, while communication delays between nodes belonging to Network 2 become large. It is because that they do not wake up at the same time any more for different operational frequencies as shown in Figure 9. The reason that communication delay between Node (2-1) and Node (2-2) is small in the case of “proposal” is that both Node (2-1) and Node (2-2) are located at the border and they operate at similar operational frequency. As stated above, communication delay results from waiting in transmission, during which a node consumes energy. We next evaluate energy consumption, which is a major concern of a wireless sensor network.

**Figure 12.**
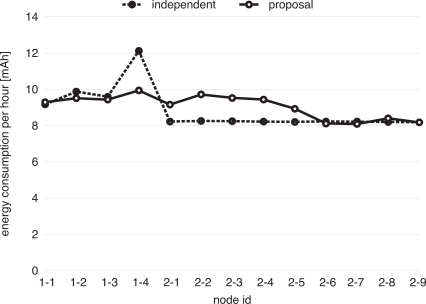
Energy consumption per hour.

Then, simulation results on energy consumption are summarized in Figure 12. As shown in Figure 12, in the case of “independent”, Node (1-4), which is located at the border of Network 1, consumes the largest energy, which causes energy dissipation at the border. On the other hand, in the case that our proposal is adopted, the amount of energy consumed at the border node is reduced from 12.1 mAh to 9.9 mAh at the sacrifice of increased energy consumption at nodes in Network 2. In the case that interconnection is mediated by a gateway node [9], it consumes as much energy as a border node of “independent” in Figure 12. Although the total amount of consumed energy is larger with our proposal than the case of “independent”, we consider that our proposal benefits wireless sensor networks in balancing energy consumption among nodes, which leads to prolongation of the lifetime of border nodes.

### 4.4. Robustness against Loss of Control Messages

**Figure 13.**
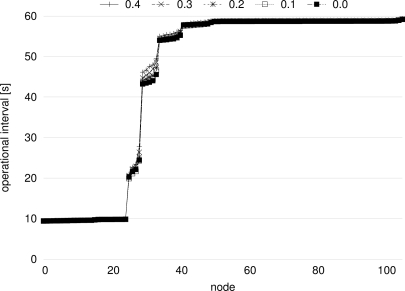
Operational interval with different packet loss probability.

In order to confirm that our proposal accomplishes the stepwise synchronization under the influence of loss of control messages, we dropped stimulus messages at the constant probability. Figure 13 shows the average operational interval during the last 10,000 s of simulation time averaged over 10 simulation runs for each of message loss probability of 0.0 (no loss), 0.1, 0.2, 0.3, and 0.4. In Figure 13, Node 0 through 24 belong to Network 1 and the others belong to Network 2. In Figure 13, we can see that the stepwise synchronization is accomplished despite the loss probability of stimulus messages. In the figure, the operational interval of nodes near the border of Network 2 becomes slightly larger as the loss probability increases, but operational intervals moderately change in a stepwise fashion nevertheless.

### 4.5. Adaptivity to Difference of Operational Frequencies of Networks

**Figure 14.**
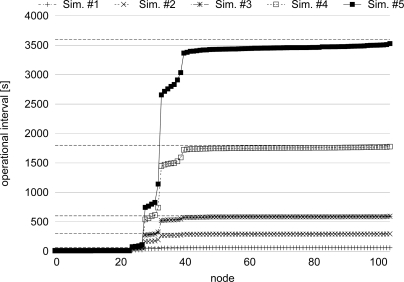
Operational interval with different operational frequency.

In order to see how the stepwise synchronization is accomplished among networks with largely different operational frequencies, we changed the operational frequency of Network 2 as shown in Table 3 and conducted 10 simulation runs for each of operational frequency settings. *A_b_* and *A_ε_* are set at 0.7 and 0.4, respectively. Figure 14 shows the average operational interval during the last 10,000 s of simulation time averaged over 10 simulation runs. In Figure 14, dashed lines show original operational intervals of nodes in Network 2 for each of settings. In Figure 14, the stepwise synchronization is accomplished independently of difference in operational frequencies. For example, in the case of the 5th simulation setting, where the difference of operational frequencies is largest and the operational frequency of Network 1 is about 360 times faster than that of Network 2, all border nodes of Network 2, *i.e.*, nodes from Node 25 to Node 28, change their operational interval from 3,600 s to about 80 s. The operational interval of nodes around those border nodes become about 800 s. As the distance to the border increases, the operational interval of node becomes longer. In Figure 14, although the operational interval of nodes distant from the border in Network 2 also becomes slightly shorter than the original operational interval, these nodes can keep their operational interval close to the original operational interval to prevent the increase of the energy consumption incurred by the frequent operation.

### 4.6. Influence of Parameter Settings

**Figure 15.**
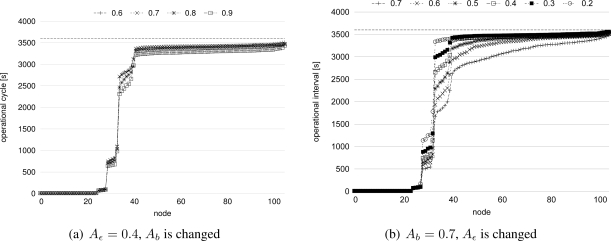
Operational interval (*A_b_* or *A_ε_* are changed).

Attenuation coefficients *A_b_* and *A_ε_* influence the degree that distant nodes are stimulated by other network. In Figure 15, we change either of *A_b_* and *A_ε_* while the other parameter is fixed. Operational intervals are set as #5 in Table 3. As shown in Figure 15(a), *A_b_* does not influence the stepwise synchronization much. *A_b_* affects the amount of the dissipation parameter *b*, and *b* becomes larger as *A_b_* increases. In our stepwise synchronization, a node receives a stimulus message in a short duration before it fires due to the duty cycling. Therefore, whenever a node receives a stimulus message, the phase of the node is large. In the region where the phase is large, the effect of the difference of the dissipation parameter *b* on the phase-state function is not large as shown in Figure 1. On the contrary, as *A_ε_* increases, the degree that nodes change their operational frequency becomes larger as shown in Figure 15(b). It is because that *A_ε_* affects the amount of the phase shift on receiving a stimulus message, *i.e.*, the amount of the stimulus *ε*, and the stimulus *ε* becomes larger as *A_ε_* increases. When a node receives a stimulus message, the phase of the node reaches 1 form 0 in a shorter duration with larger *ε* and the operational interval of the node becomes shorter as shown in Figure 6. It is obvious that large *A_ε_* involve further nodes in stepwise synchronization. By being involved in stepwise synchronization, a node operates at shorter intervals and the frequent operation consumes larger energy. We consider that we should set *A_ε_* at 0.3 or 0.4 to accomplish a stepwise synchronization, where only nodes located near the border adjust their operational frequency to bridge the gap in their intrinsic operational frequency while the remaining nodes keep their frequency closer to their intrinsic frequency.

In conclusion, independently of difference in operational frequencies, we can adopt the same pair of *A_b_* = 0.7 and *A_ε_* = 0.4 to accomplish the stepwise synchronization. We should note here that stepwise adaptation of operational frequencies is limited to the area near border nodes and the number of nodes does not affect the stepwise synchronization or parameter setting.

## 5. Conclusions

In this paper, to achieve smooth and moderate inter-networking between wireless sensor networks with different operational frequencies, we propose a stepwise synchronization-based inter-networking mechanism. In this mechanism, we adopt the pulse-coupled oscillator model to autonomously accomplish the stepwise synchronization. Through simulation experiments, it was shown that the delay in communication between border nodes and the energy consumption at the border nodes were reduced, but at the sacrifice of energy at nodes near the border in the slower network. Although the total amount of consumed energy is larger with our proposal than the case where both networks keep their intrinsic operational frequencies, we consider that our proposal benefits wireless sensor networks in that (i) it balances energy consumption among nodes, which leads to prolongation of the lifetime of wireless sensor networks, (ii) it can enable wireless sensor networks with largely different operational frequencies to synchronize with each other, and (iii) since the stepwise synchronization emerges as a consequence of mutual interaction between nodes and there is no deterministic rule to determine stepwise operational frequencies, it can adapt to various situations, e.g., increase in the number of networks to cooperate, cooperation among moving networks, and different degree of cooperation.

Although we verified that stepwise synchronization can be accomplished without fine-tuning of parameters in the evaluated scenarios, we consider that tuning of parameters are helpful for faster and stable synchronization. Furthermore, other conditions such as the number of networks would affect synchronization. Regarding the speed, synchronization takes time because it is self-organized. As such, our proposal cannot be directly applied to scenarios where nodes have the high mobility. We further plan to evaluate the proposal in such scenarios where there are more than three networks to cooperate and the degree of overlapping—the number of border nodes—changes dynamically.

## Figures and Tables

**Table 1.** Parameter settings.

PCO	

**parameter**	**value**
*b_max_*	3.0
*ε_max_*	0.1
*b_min_*	1.0
*ε_min_*	0.02
*A_b_*	0.7
*A_ε_*	0.4

X-MAC	

**parameter**	**value** [ms]
*S_pre_*	1.0
*S_ack_*	1.0
*S_stim_*	1.0
*S_data_*	3.0
*R_s_*	200
*R_l_*	20

**Table 2.** Energy consumption model.

**parameter**	**value**
Initial energy	2,000 mAh
Processor active current	8 mA
Sleeping current	15 uA
Sending current	9.9 mA
Waiting and receiving current	19.7 mA

**Table 3.** Operational frequency settings.

	**operational frequency [Hz]**	**(operational interval [s])**

	**Network 1**	**Network 2**
#1	0.1 (10)	0.017 (60)
#2	0.1 (10)	0.0033 (300)
#3	0.1 (10)	0.0017 (600)
#4	0.1 (10)	0.00055 (1,800)
#5	0.1 (10)	0.00027 (3,600)
